# From transporter to transceptor: Signaling from transporters provokes re-evaluation of complex trafficking and regulatory controls: Endocytic internalization and intracellular trafficking of nutrient transceptors may, at least in part, be governed by their signaling function

**DOI:** 10.1002/bies.201100100

**Published:** 2011-11

**Authors:** Johan Kriel, Steven Haesendonckx, Marta Rubio-Texeira, Griet Van Zeebroeck, Johan M Thevelein

**Affiliations:** 1) Laboratory of Molecular Cell Biology, Institute of Botany and MicrobiologyK. U. Leuven, Leuven, Belgium; 2) Department of Molecular MicrobiologyVIB, Leuven-Heverlee, Flanders, Belgium

**Keywords:** intracellular trafficking, nutrient sensing, nutrient transport, signal transduction, transceptor

## Abstract

When cells are starved of their substrate, many nutrient transporters are induced. These undergo rapid endocytosis and redirection of their intracellular trafficking when their substrate becomes available again. The discovery that some of these transporters also act as receptors, or transceptors, suggests that at least part of the sophisticated controls governing the trafficking of these proteins has to do with their signaling function rather than with control of transport. In yeast, the general amino acid permease Gap1 mediates signaling to the protein kinase A pathway. Its endocytic internalization and intracellular trafficking are subject to amino acid control. Other nutrient transceptors controlling this signal transduction pathway appear to be subject to similar trafficking regulation. Transporters with complex regulatory control have also been suggested to function as transceptors in other organisms. Hence, precise regulation of intracellular trafficking in nutrient transporters may be related to the need for tight control of nutrient-induced signaling.

## Introduction

Extracellular nutrients can trigger induction of transporters and enzymes required for their uptake and metabolism. There are, however, also numerous examples of transporters that are more highly expressed in the absence of their substrate. Because of the higher transport capacity, higher affinity, and/or broader substrate range of these transporters, the cells are thought to be better equipped for taking up minute amounts of substrate from the environment compared to cells with a regular transporter profile. Interestingly, when the substrate becomes available again in adequate quantities, the cells use elaborate mechanisms to rapidly remove the transporter from the plasma membrane and sort it to the vacuole/lysosome for destruction. Apparently, the continued presence of the transporter at the cell periphery in the presence of abundant substrate is in some way detrimental to the cells, necessitating its specific recognition, removal, and destruction. Up to now, this observation has been interpreted only in light of excessive uptake of substrate that may result from a high level of the transporter in the membrane.

**Figure 1:**
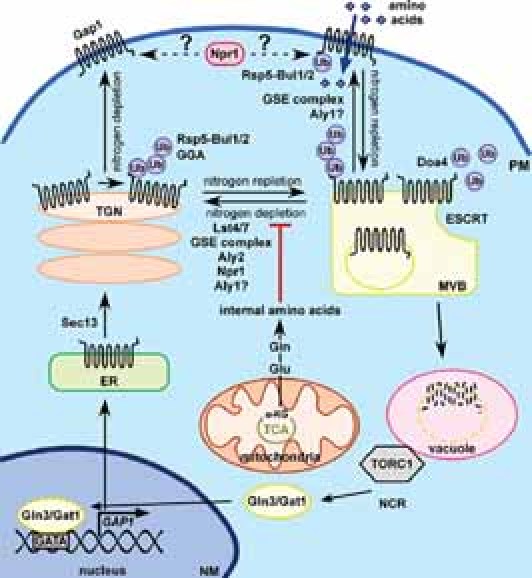
The complex intracellular trafficking pathway of the Gap1 amino acid transceptor as influenced by the nitrogen supply. Newly synthesized Gap1 is transported through the secretory pathway from the endoplasmic reticulum (ER) to the trans-Golgi network (TGN), from where it can be sorted to either the plasma membrane under nitrogen-depleted conditions, or to the vacuole/lysosome for subsequent degradation under favorable conditions of nitrogen supply. Vacuolar sorting is mediated first by the Rsp5-Bul1/2 ubiquitin ligase complex, with cooperation from the GGA coat proteins functioning as ubiquitin-sorting receptors at the TGN. In the next step, ubiquitinated Gap1 is delivered to the multivesicular body (MVB), where the second sorting decision is made: the ubiquitinated permease may either become MVB cargo for delivery to the vacuole/lysosome, or may be rerouted to the plasma membrane. Recycling of Gap1 can occur directly from the MVB to the plasma membrane in a GSE complex (GTPase-containing complex for Gap1 sorting in the endosomes) assisted trafficking step, or indirectly via the TGN, which involves Lst4/7, the GSE complex, the protein kinase Npr1 and the α-arrestin Aly2. Aly1 is proposed to function independently of known recycling mediators. Elevated internal amino acid levels, especially glutamate and glutamine, block the MVB-to-TGN recycling of Gap1. Formation of inwardly budding MVB vesicles is regulated by sequential participation of the ESCRT protein complexes. Before Gap1 is endocytosed into the MVB, ubiquitin is removed by the deubiquitination enzyme, Doa4. Subsequently, direct fusion of the MVB with the vacuole/lysosome results in the delivery of Gap1-containing internal MVB vesicles to the vacuolar lumen for proteolytic degradation. The Npr1 protein kinase is required for stabilization of Gap1 at the plasma membrane, but its precise action mechanism has remained unclear. Transcription of the *GAP1* gene is under nitrogen catabolite repression (NCR) control, i.e. the gene is expressed in the presence of poor or no nitrogen sources and it is repressed in the presence of good nitrogen sources. Under conditions of nitrogen depletion or inhibition of TORC1 by rapamycin, Gln3 and Gat1 localize to the nucleus where they bind to GATA sequences within the promoter of *GAP1* to stimulate transcription. The information in this figure has been compiled from the following references, in which more detailed information on the different components involved or possibly involved in Gap1 trafficking can be found: [3, 48]^64^–[68].

The Gap1 general amino acid permease in the yeast *Saccharomyces cerevisiae* has been studied as a model system for substrate-regulated intracellular trafficking. Research on this protein has revealed a complex set of regulatory mechanisms, not only controlling amino acid-induced endocytic internalization, but also governing control of Gap1 secretion to the plasma membrane as well as intracellular trafficking by the quality and quantity of extracellular amino acids [1–5] (Fig. 1). Growth on poor nitrogen sources or complete nitrogen deprivation causes strong induction of *GAP1* at the transcriptional level and maximal accumulation of the protein at the plasma membrane [1]. Addition of a good nitrogen source triggers rapid ubiquitination, endocytic internalization, and sorting to the multivesicular body (MVB) and the vacuole/lysosome where the protein is degraded [4, 5]. Interestingly, the Gap1 protein en route from the Golgi apparatus to the plasma membrane is also affected by the presence of external amino acids. The Gap1-containing secretion vesicles released by the trans-Golgi network (TGN) are deviated to the MVB and sorted to the vacuole/lysosome [2, 3].

The *GAP1* gene is only expressed during growth on poor nitrogen sources and under conditions of nitrogen starvation. Amino acid addition triggers rapid repression [1]. However, transcriptional regulation is apparently either too slow, or for other reasons not adequate enough, to allow proper adjustment of the level of Gap1. The complex post-translational controls on the intracellular trafficking of Gap1 have raised many questions as to why this specific amino acid transporter, which is only one of about 20 amino acid transporters in the yeast plasma membrane, needs such a complex regulation.

Although studied in less detail, similar substrate-induced trafficking controls have been reported for multiple other transporters in yeast. Examples include the Fur4 uracil permease [6], Pho84 phosphate permease [7], and several permeases for metal ions [8–12] and siderophores [13, 14]. All these transporters are strongly induced in the absence of their substrate, and addition of substrate causes their rapid removal from the plasma membrane.

**Figure 2:**
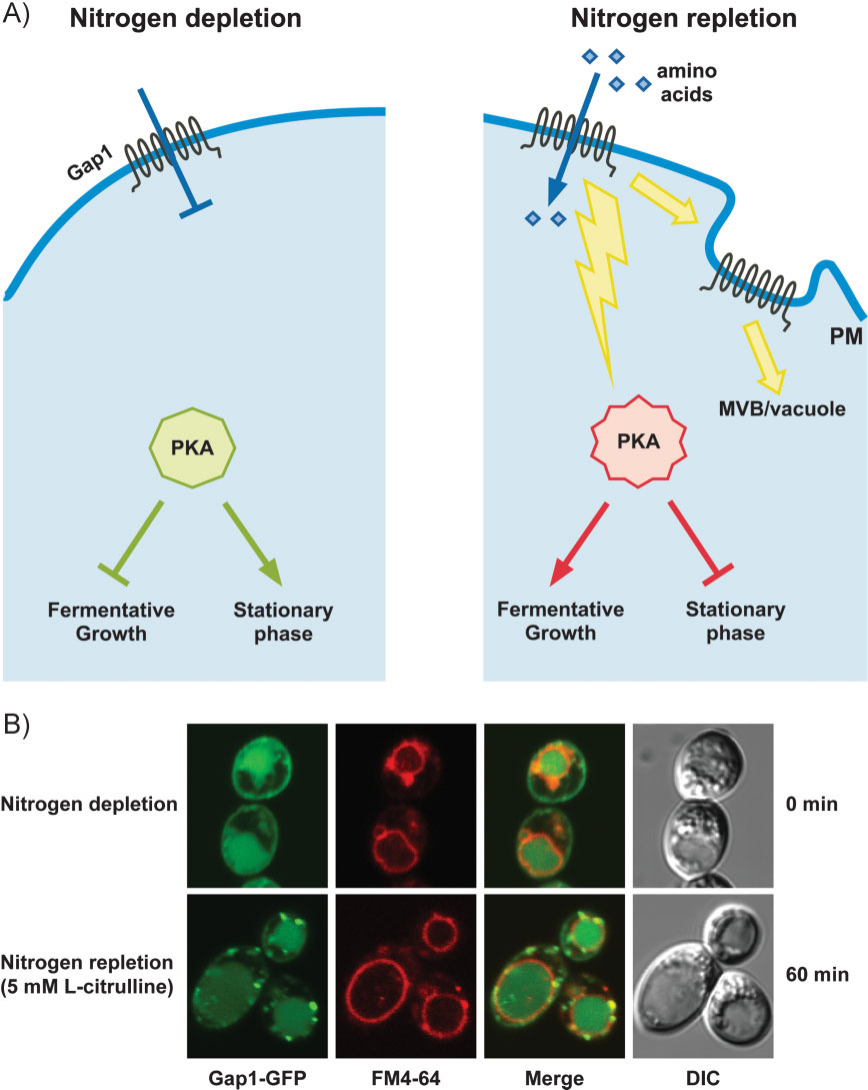
The signaling function and intracellular location of the Gap1 amino acid transceptor upon resupply of an amino acid to nitrogen-starved cells. **A:** When faced with nitrogen deprivation, yeast cells enter into a quiescent, resting phase, known as stationary phase. Under these conditions, PKA has low activity and cellular characteristics correlated with fermentative growth are down-regulated, whereas stationary-phase characteristics are up-regulated. The amino acid transceptor Gap1 is stabilized at the plasma membrane. Addition of amino acids to nitrogen-depleted cells causes rapid activation of PKA, which helps preparing the cells for both the resumption of fermentative growth and the down-regulation of stationary-phase characteristics. Amino acid-induced activation of PKA was shown to be mediated by the receptor function of Gap1. Deciphering the downstream signaling pathway connecting the amino acid transceptor to PKA, requires further research. Amino acid uptake by Gap1 also results in its ubiquitination, endocytic internalization, and targeting to the vacuolar degradation pathway. The rapid, nitrogen-induced down-regulation of Gap1 may have evolved as an additional regulatory mechanism, safeguarding the cell against the detrimental effects of overstimulation of the PKA pathway. **B:** The fate of Gap1-GFP is shown as a function of the external nitrogen supply. Under conditions of nitrogen depletion (24 hours), Gap1-GFP is localized at the plasma membrane and in the vacuolar lumen. Addition of 5 mM l-citrulline as sole nitrogen source for 60 minutes results in delocalization of Gap1-GFP from the plasma membrane to intracellular vesicles and especially to the vacuole/lysosome. The cells were simultaneously co-stained with the lipophylic dye FM4-64, which after endocytosis, locates at the vacuolar limiting membrane.

More recent work has uncovered an additional signaling role for some of the transporters regulated in this way. When yeast cells are deprived or limited for an essential nutrient, they not only induce specific transporters, but also reduce their growth rate, which makes the cells acquire a range of phenotypic properties typical for slow-growing or stationary-phase cells. These include accumulation of the reserve carbohydrate glycogen and the reserve and stress protection sugar trehalose, acquirement of high stress tolerance (among other reasons because of induction of chaperone proteins), down-regulation of ribosomal protein gene expression, and increased cell wall resistance. The pathway involved in controlling these traits as a function of nutrient availability is the protein kinase A (PKA) pathway [15]. Low activity of the pathway results in the same phenotypes as observed in starved cells, while an overactive PKA pathway prevents the establishment of stationary-phase characteristics. The highest activity of the PKA pathway is observed in cells growing rapidly on glucose using ethanolic fermentation, while any reduction in the growth rate of such cells by limitation or deprivation of an essential nutrient, causes lower activity of the pathway. Although the molecular link between nutrient availability/growth rate and the activity of the PKA pathway is only partially understood, several specific nutrient sensors responsible for rapid activation of the PKA pathway upon re-addition of the limiting nutrient have been identified. Interestingly, all these nutrient sensors are transporters that are strongly induced under conditions of substrate deprivation or limitation. The functioning of these transporters as receptors has been documented in greatest detail for the Gap1 general amino acid permease [16, 17] (Fig. 2), the Pho84 phosphate permease [18, 19], and to a lesser extent for the Mep1,2 ammonium permeases, although the latter have not been reported to undergo rapid ammonium-induced endocytic internalization [20]. The dual function as both transporter and receptor has led to the novel concept of “transceptor” [21]. Similar bifunctional transceptor proteins have, since then, also been proposed in other organisms, including plants and mammals [22–25].

## Transporters were discovered to act as receptors: “Transceptors”

Addition of amino acids to nitrogen-deprived yeast cells triggers rapid changes in multiple targets of the PKA pathway, the most rapid being a fivefold increase in the activity of the trehalase enzyme due to post-translational phosphorylation by PKA. All these rapid changes are eliminated upon deletion of *GAP1* [16]. Since Gap1 is a major amino acid carrier in nitrogen-starved cells, this might have been an indirect effect of the strong reduction of amino acid import into the cells. However, many results have been obtained indicating that Gap1 itself acts as an amino acid sensor, which signals to the PKA pathway [16]. l-Citrulline triggers signaling when transported by Gap1, but not when transported by other amino acid carriers. Specific point mutations in Gap1 differentially affect transport and signaling, and two C-terminally truncated Gap1 alleles have been obtained that cause constitutive activation of the PKA pathway. Abolishing metabolism of the transported amino acid does not prevent signaling, consistent with the observation that non-metabolized amino acid analogs, including d-amino acids, transported by Gap1 also trigger signaling. Recent work has identified amino acid analogs that are not transported but are still able to trigger signaling and thus cause Gap1 to function as a true receptor protein [17]. On the other hand, not all amino acid analogs that bind to the amino acid binding site of Gap1 trigger signaling, indicating that the substrate/ligand has to induce a specific conformational change in the transceptor, reminiscent again of the action of ligands on true receptors. Substituted cysteine accessibility method (SCAM) analysis has revealed that Gap1 uses the same amino acid binding site for transport and signaling. Whether substrates can be transported without triggering signaling is unclear. Recent work has identified multiple deletion strains in putative Gap1 interacting proteins in which transport and signaling were differentially affected [26]. This clearly shows that the two functions of the transceptor can be separated. Recent work has shown that of the six Gap1 homologs in *C. albicans*, three can also function as transceptors for rapid amino acid activation of the PKA pathway when expressed in *S. cerevisiae* [27].

How Gap1 signals to PKA is unclear. It seems to use an unconventional mechanism since signaling is not associated with an increase in the cAMP level, and is even observed in strains lacking the regulatory subunit of PKA and thus unable to respond to changes in the cAMP level [28, 29]. The observation that non-transported amino acid analogs can act as agonists for signaling argues against any transport-associated process, such as proton symport, as trigger for the signaling event [17].

Very similar results have been obtained for the Pho84 phosphate transporter, which functions as transceptor for rapid activation of the PKA pathway in phosphate-deprived cells [18, 19]. Glycerol-3-phosphate, although not transported by Pho84, triggers rapid Pho84-dependent activation of the PKA pathway when added to phosphate-starved cells. Glycerol-3-phosphate is taken up by the Git1 carrier in yeast, but transport through this carrier does not trigger rapid activation of the PKA pathway. Several other non-transported phosphate-containing compounds triggering signaling were found, but as in the case of Gap1 not all compounds that interact with the substrate-binding site in Pho84, are able to trigger signaling. The same phosphate-binding site in Pho84 was also shown to be involved both in transport and signaling. These observations support the concept that the substrate-ligand has to induce a specific conformational change to trigger signaling, as is the case in classical receptors.

Ammonium-induced activation of the PKA pathway in nitrogen-starved cells is mediated by the ammonium transceptor Mep2 and to a somewhat lower extent by Mep1 [20]. Specific alleles of Mep2 have been obtained that were differentially affected in transport and signaling, and the non-metabolizable ammonium analog, methylammonium, also triggers signaling. Whether the Gap1 and Mep1,2 transceptors employ the same signaling mechanism is unclear, but in both cases the signaling is dependent on the Sch9 protein kinase [20, 30].

Yeast cells also possess three transporter-like proteins in the plasma membrane that lack any detectable transport function: the glucose sensors Snf3 and Rgt2 [31], and the amino acid sensor Ssy1 [32–34]. They do not seem to have a pleiotropic effect on cellular regulation like the transceptor's signaling to the PKA pathway, but rather control more specific signaling pathways that regulate the expression of regular glucose or amino acid transporters, respectively. A mechanistic model has been proposed for these sensors [35, 36]. Whether all transporter-like nutrient sensors function mechanistically in a similar way is currently unclear.

## Level and trafficking of the Gap1 transceptor are under complex control

### The *GAP1* gene is expressed under conditions of no or poor nitrogen supply

The *GAP1* gene is controlled by nitrogen catabolite repression (NCR), which regulates expression of genes as a function of the nitrogen status of the medium [37–39]. In nitrogen-deprived cells or in cells growing on poor nitrogen sources, like proline, *GAP1* is strongly induced and its accumulation at the plasma membrane creates a high-affinity, high-capacity uptake system for amino acids. Gap1 is a highly promiscuous carrier that transports a wide variety of amino acids, including not only the regular protein amino acids but also non-metabolizable amino acids like d-amino acids [1, 40]. Because of its expression in nitrogen-starved cells, its high affinity and its substrate promiscuity, Gap1 has primarily been envisioned as an amino acid scavenger. It appears logical to consider Gap1 as a high-affinity transporter for scavenging low levels of external amino acids under nitrogen-limited conditions and the other amino acid permeases as low-affinity transporters for uptake of amino acids under plentiful conditions. However, there is no such clear distinction in yeast between the expression of high- and low-affinity amino acid permeases. For instance, *AGP2* and *AGP3* encode low-affinity permeases, able to transport multiple amino acids, and are expressed under conditions of nitrogen limitation [41]. *BAP2* encodes a high-affinity branched-chain amino acid permease expressed under nitrogen-rich conditions [42]. *GNP1* encodes a high-affinity permease for glutamine, asparagine, and other amino acids [43], while *DIP5* encodes a high-affinity permease for glutamate, aspartate, and other amino acids [44]. Both transporters are expressed on both rich and poor nitrogen sources.

Also, the uptake by Gap1 of non-metabolizable amino acids and analogs raises questions concerning the physiological relevance of such a promiscuous transport function, especially since Gap1 appears to be the only carrier in yeast able to take up these non-metabolizable nitrogen compounds. What could be the physiological purpose of expressing a transporter that takes up so many useless and even toxic compounds? Moreover, since Gap1 is expressed only under conditions of poor nitrogen availability, one wonders why it transports non-metabolizable amino acid analogs that act as competitive inhibitors for the uptake of regular, metabolizable amino acids under conditions where the cells are in acute need of metabolizable nitrogen.

The predominant existence of a single promiscuous amino acid transporter in nitrogen-limiting conditions appears to be more compatible with its function as a transceptor [45]. If a major role of Gap1 is to detect the presence of amino acids and other potential nitrogen sources in the medium, and to activate the cellular machinery for rapid stimulation of the initiation of protein synthesis and fermentation, the substrate promiscuity of Gap1 for non-metabolizable amino acid analogs makes more sense. Indeed, when cells in their natural setting experience a new nutrient condition, it will in all likelihood contain a mixture of nutrients of variable quality. Hence, if the yeast cells detect non-metabolizable amino acid analogs in their environment, the chance that metabolizable amino acids are also present, is very high. Given the extreme competition for food in the microbial world, it may be more useful for yeast cells to immediately activate the cellular machinery for a possible rapid increase in growth and fermentation, rather than to wait until the presence of metabolizable amino acids is sure. If not, these nutrient sources may be consumed by competing microorganisms. When it turns out that no metabolizable nitrogen sources are present, the PKA pathway and the growth machinery will be down-regulated again, since it has been shown that persistent activation requires active metabolism [15].

### Amino acids induce endocytic internalization of Gap1

As soon as a regular supply of one or more amino acids becomes available again, Gap1 is ubiquitinated, internalized, degraded, and substituted by a range of other amino acid carriers, of which the induction is triggered by the non-transporting amino acid sensor Ssy1 [46, 47]. The expression of a range of amino acid carriers with different specificities under nitrogen-rich conditions probably allows for better fine-tuning of the amino acid uptake to the requirements of protein synthesis and metabolism. One specific reason for expression of multiple amino acid carriers may be that, if only one single carrier was present, a large excess of a single amino acid would prevent uptake of all other amino acids by competitive inhibition. Intracellular amino acid catabolism and interconversion can, to a certain extent, overcome this problem, but may have been evolutionary less efficient than simply dividing the uptake of the appropriate amino acids over multiple carriers with different specificities.

The mechanism of amino-acid induced Gap1 internalization has been studied in detail (Fig. 1). The protein is thought to be protected from internalization by phosphorylation, which is indirectly dependent on the Npr1 protein kinase [48]. Transport of an amino acid triggers ubiquitination, possibly preceded by dephosphorylation, and followed by endocytic internalization, sorting to the MVB and degradation in the vacuole [4, 5]. Whether amino acid transport through Gap1 is required or whether only recognition of the amino acid by binding to Gap1 is sufficient to trigger ubiquitination and/or endocytic internalization, is not clear. Conversely, whether a substrate can be transported without triggering ubiquitination and endocytic internalization is also not known.

Rapid inactivation of Gap1 after arrival of amino acids appears to be very important for the cells since Gap1 undergoes an additional rapid inhibition process, which was clearly observed with the internalization-defective Gap1^K9R, K16R^ allele [49]. It required active transport through Gap1 and not merely the presence of extra- or intracellular amino acids. It was suggested to be due to a reversible modification or an allosterically induced conformational change, causing reduced inherent activity or modified interaction with one or more inhibitory proteins. Given that the receptor function of Gap1 can be activated by non-transported amino acid analogs [17], the question arises whether the binding of an amino acid to Gap1 may be enough to trigger this regulatory inhibition of its transport function. Since transport is not required for signaling, the latter may also not be subject to the reversible inhibition process.

Risinger et al. [49] also demonstrated that cells expressing the internalization-defective Gap1^K9R, K16R^ allele were highly sensitive to addition of single l-amino acids, causing loss of cell viability. Complex amino acid mixtures were not toxic at all. Excessive uptake of a single amino acid, causing a gross excess in the intracellular level of this amino acid, was suggested to result in toxicity through possible tRNA synthetase mischarging and concomitant protein malfunctioning.

These results are intriguing since intracellular hyperaccumulation of single amino acids has been observed before in different yeast mutants. This was never reported to result in toxicity. For instance, strains with deficient feedback inhibition of aspartate kinase accumulate up to 40 times more threonine than the wild-type strain, without apparent loss of cell viability [50]. Secretion of specific amino acids by yeast cells is usually due to internal hyperproduction of the amino acid, and such strains have been exploited as cell factories for amino acid production [51]. Strong accumulation of proline and arginine were even shown to have beneficial effects to the stress tolerance of yeast [52–54]. This suggests that the single amino acid toxicity may not simply result from an extreme imbalance in the intracellular pools of amino acids. It should be noted, however, that the use of rich growth media may explain the discrepancies in cell viability with the study by Risinger et al. [49].

Ligand-induced endocytic internalization of receptor proteins is well established and generally considered to act as a mechanism to prevent overstimulation of signaling pathways [55]. The same may apply to the Gap1 transceptor. Overstimulation of the PKA pathway is toxic to yeast cells under conditions of no or slow growth [56–59], and has been reported to trigger apoptosis [60]. Hence, rapid removal of Gap1 from the plasma membrane may down-regulate amino-acid induced stimulation of the PKA pathway. Interestingly, a C-terminally truncated Gap1 allele, which acted as a constitutively activating allele on the PKA pathway, completely inhibited growth of the cells on a non-fermentable carbon source, clearly showing that overactive Gap1 is detrimental to the cells [16]. Hence, the toxicity caused by single amino acids in cells expressing internalization-defective Gap1^K9R, K16R^ may be due to interference with down-regulation of the Gap1 signaling function.

### Extracellular amino acids control intracellular trafficking of Gap1

The Gap1 transceptor is not just secreted through the secretion pathway to the plasma membrane and internalized by endocytosis for delivery and breakdown in the vacuole. The cells also appear to maintain a pool of internally recyclable Gap1 that can be rapidly sent to the plasma membrane or diverted to the vacuole depending on the extracellular amino acid conditions (Fig. 1). The presence of good quality nitrogen sources in the environment, as well as an increase in external/internal amino acid levels, normally promote vacuolar sorting of both the plasma membrane and the TGN pools of Gap1 [61]. However, other research revealed the puzzling existence of internal pools of Gap1 in the presence of reasonably good nitrogen sources, such as glutamate [2]. Why it is so important to maintain internal pools of this transporter and to protect a fraction of the protein from vacuolar degradation has become an important question in this field. From an energetic viewpoint, constitutive cycling of membrane proteins is an energy-expensive process because of the GTP and ATP required in endo- and exocytosis. Similar to that proposed for futile metabolic cycles, the energetic cost may be the necessary expense for running an extremely sensitive system [62]. A balance between external and internal levels of amino acids seems to dictate the final fate of internal Gap1 [63]. This balance is determined, in particular, by the cellular content of glutamate and glutamine. Mutants with enhanced glutamate and glutamine levels display constitutive vacuolar sorting of Gap1, while those with reduced levels display constitutive sorting of Gap1 to the plasma membrane.

Recycling of Gap1 from endosomes to the plasma membrane was first noticed in mutants with defects in the machinery (ESCRT: endosomal sorting complex required for transport) responsible for recognition and sorting of cargo proteins to the vacuole. Such mutants exhibited enhanced levels of Gap1 at the plasma membrane, indicating that failure in sorting to the vacuole resulted in increased recycling to the plasma membrane [3, 64]. Several proteins and protein complexes have been identified that play a role in the control of intracellular Gap1 sorting, suggesting that the process is for some reason tightly controlled (Fig. 1) [3, 48, 64–68].

There are other examples of transporters, such as the mammalian GLUT4 glucose carrier, that are maintained in intracellular vesicles [69]. However, these carriers are generally recruited to the plasma membrane when the external supply of their substrate is high, rather than low as is true for Gap1. A first possible explanation is that microbes like yeast experience dramatic up- and down-shifts in their supply of nutrients and that therefore Gap1 is kept in reserve as a scavenger transporter for times when the nitrogen supply suddenly drops. A second explanation may be that Gap1 is needed to sense external amino acid levels for control of the PKA pathway, and that a sudden drop in the intracellular amino acid level raises questions about the precise nitrogen status in the extracellular medium. Rapid recycling of Gap1 to the plasma membrane could be a means to check the external amino acid level and adjust activation of the PKA pathway accordingly, resulting in proper regulation of a multitude of cellular properties related to growth and fermentation. Prevention of Gap1 recycling to the plasma membrane in the presence of abundant amino acids could again be a way to prevent overstimulation of the PKA pathway. In principle, the intracellular pool of Gap1 could be used to sense intracellular levels of amino acids, but since the exogenous side of Gap1 is located within the lumen of the endosomes, this appears less likely. The maintenance of Gap1 in an intracellular pool may be related in an alternative way to its signaling function. For several receptors in mammalian cells, it has been demonstrated that instead of terminating their signaling after endocytosis, they continue to signal and modify their transducer activity as endosome residents [70]. Whether the Gap1, Mep2, or Pho84 transceptors are able to maintain signaling to the PKA pathway, once internalized, is unknown. What is well established, however, is that after the internalization of the transceptors the cells maintain high PKA activity during subsequent fermentative growth [15]. Whether the internalized transceptors, for instance as part of a separate, protected pool, play a role in keeping PKA activity high is unclear. One argument that may support this hypothesis is that yeast strains with elevated PKA activity display reduced levels of Gap1, apparently through a post-transcriptional mechanism [71], which would be consistent with feedback inhibition of PKA on one of its nutrient inducers. More elaborate work is required, however, to further elucidate the post-transcriptional connection between PKA and Gap1 levels and/or transport and signaling activity.

**Figure 3:**
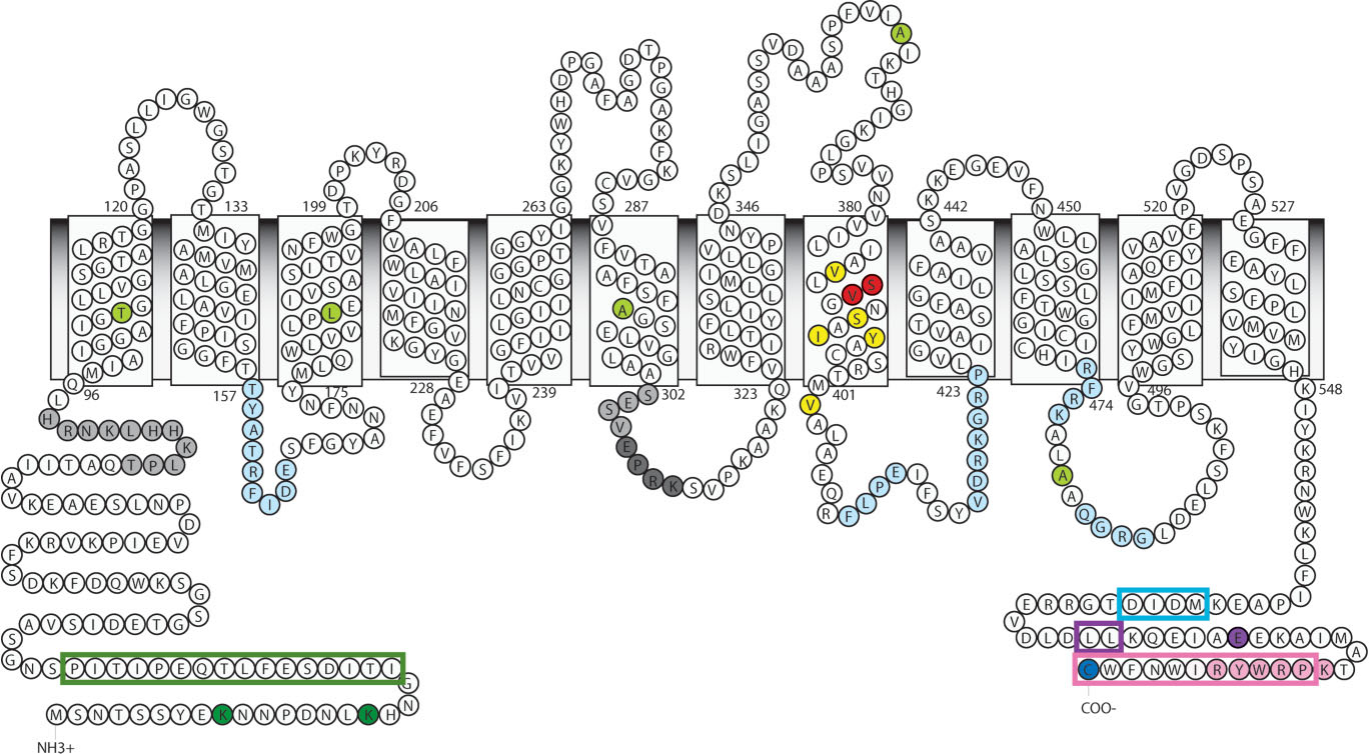
Structural features of the Gap1 transceptor important for transport, signaling, or intracellular trafficking. Gap1 is a protein with 602 amino acid residues, arranged in 12 transmembrane domains (TMD), with cytosolic N- and C-termini. Two main ubiquitination acceptor residues, lysines K9 and K16 (dark green), are located at the N-terminus. Neighboring residues 20–35 (framed in dark green) are essential for ubiquitination of these lysines. Additional residues in the N-terminus or within the middle internal cytosolic loop and located near a TMD, are important for transport but not for localization of Gap1, (light gray). Several domains in intracellular loops are important for Gap1 exit from the ER (light blue). An EPRK sequence (dark gray), located in the third cytosolic loop, is crucial for proper sorting of Gap1. Important features have also been discovered within the TMDs of Gap1. A consensus amphipathic region (CAR) has been identified in TMD8, containing several residues that are essential for both transport and signaling (yellow). Among these, residues S388 and V389 (red) were shown to be part of the amino acid-binding site involved in both transport and signaling. Additional residues positioned within TMDs or external/internal loops, and essential for transport of specific groups of amino acid substrates, are: T106 (TMD1), L185 (TMD 3), A297 (TMD6), A365 (extracellular loop between TMD7 and 8), and A479 (intracellular loop between TMD10 and TMD11 (light green). The C-terminus is important for secretion of Gap1 to the plasma membrane. It contains a Sec23/Sec24 COPII recognition motif, involved in ER exit (framed in light blue), a di-leucine motif (framed in purple), and a glutamate residue (purple) in a predicted α-helix, the latter essential for endocytosis of Gap1. Deletion of the last 11 amino acids in the C terminus (Gap1ΔC2, framed in pink) perturbs ubiquitination and endocytosis. Mutagenesis of E583 (purple) blocks both polyubiquitination and direct sorting of Gap1 from the Golgi to the vacuole. The small GTPase, Gtr2, involved in Gap1 recycling, also binds to the C-terminus. This is dependent on a tyrosine-containing motif, KPRWYR (pink), near the end of the C-terminus. The last three residues in the C-terminus, FWC, are highly conserved among yeast amino acid permeases. The cysteine of this tripeptide (dark blue) is palmitoylated by the Pfa4 palmitoyl transferase. Truncation of the last 14 or 26 amino acids from the C-terminus creates a constitutively active allele of the Gap1 transceptor that resides permanently in the plasma membrane and causes constitutive overactivation of the PKA pathway, provided it is expressed in a mutant strain that allows such truncated alleles to be secreted to the plasma membrane. The information in this figure has been compiled from the following references, in which more detailed information can be found: [4, 5, 16, 17, 46, 49, 65, 72–77].

Extensive site-directed mutagenesis and truncation analysis of Gap1 have identified amino acid residues specifically required for either ubiquitination, endocytic internalization, intracellular trafficking, signaling to the PKA pathway, or amino acid binding and recognition [4, 5, 16, 17, 46, 49, 65, 72–77] (Fig. 3). These mutant alleles will be very useful to further clarify molecular mechanisms and possible connections between these processes.

## Other transceptors and transporters in yeast also undergo complex trafficking control

Although studied in less detail, other established transceptors and candidate transceptors appear to undergo, at least in part, similar controls on their intracellular trafficking as Gap1. Addition of phosphate to phosphate-limited cells triggers rapid endocytic internalization of the Pho84 transceptor [7, 78]. This is associated with phosphorylation, probably mediated by PKA, and by ubiquitination [79]. Hence, similar to the suggestion made for Gap1, since PKA is controlled by transceptor signaling it may also be involved in feedback regulation of Pho84, including its intracellular trafficking.

Several other nutrient transporters in yeast and fungi are known to be more strongly expressed at the plasma membrane when their substrate is present in limiting levels and to undergo rapid endocytic internalization upon addition of substrate. Ubiquitination is a common signal involved in triggering endocytic internalization of membrane transport proteins under these conditions [80]. Examples that have been studied in more detail include the yeast Fur4 uracil permease [6], the metal ion transporters Smf1 [8], Zrt1 [10], Ftr1 [9], Ctr1 [12], Alr1 [11], and the siderophore transporters Arn1 [14] and Sit1 [13]. A possible transceptor function of these proteins has not been explored yet. However, these transporters are induced on medium limited for an essential nutrient and under such conditions the PKA pathway can be expected to be down-regulated. Hence, these transporters may also function as transceptors for rapid activation of the PKA pathway. Up to now, the rapid endocytic down-regulation of the Fur4 uracil permease and the other transporters has generally been interpreted as preventing overaccumulation of the substrate in the cells. Uracil, for instance, was found to be toxic to cells with high uracil permease activity [81].

For the Fur4 uracil permease [82] and the Ftr1 iron transporter [9], mutant alleles without transport activity were shown not to be internalized. This was interpreted as indicating that substrate transport through the permease was required for triggering endocytic internalization. However, similar to the lack of transport requirement for signaling by the nutrient transceptors, the question can be raised whether transport is truly required for triggering transporter internalization or whether recognition (binding) of the substrate to the transporter is enough. The Fur4 mutant allele lacking transport and internalization had a very low affinity for uracil, indicating that lack of recognition of the substrate may have been the cause of defective internalization. Also for the uric acid/xanthine transporter, AnUapA, of the filamentous ascomycete *Aspergillus nidulans*, it was shown that substrate-elicited endocytosis is dependent on transport activity [83].

Interestingly, several nutrient transporters, like the Fur4 uracil permease [84], the Tat2 tryptophan permease [85], and the maltose permease [86], are rapidly degraded upon starvation for essential nutrients like nitrogen, phosphate or carbon, or more generally upon entrance into stationary phase. Even the Pho84 transceptor is more strongly expressed under low phosphate conditions than under phosphate starvation [87]. Since it is well established that yeast strains with high PKA activity undergo apoptosis and cell death in stationary-phase or slow-growth conditions, degradation of transceptors in stationary phase may prevent aberrant overstimulation of the PKA pathway. Determination of PKA-dependent phenotypes in stationary-phase cells of strains expressing alleles of nutrient transporters that constitutively reside in the plasma membrane may reveal whether they might have the capacity to signal to the PKA pathway.

## Complex trafficking control may be indicative of transceptor functionality in other organisms

The discovery of a receptor function in several yeast transport proteins, which were previously considered to be just regular transporters, suggests that many transporters in other organisms could also be transceptors. Because the metabolism of a nutrient after its transport can affect so many parameters in a cell, any cellular change observed after the transport of a nutrient is generally ascribed to a consequence of its metabolism. Even the transport step itself may have indirect effects caused for instance by co-transported ions. Therefore, the signaling function of a transporter is very easily overlooked. Even more difficult to recognize is a situation where partial metabolism of the nutrient and transporter signaling have to act together to cause a certain cellular response. In these cases, sophisticated genetic engineering is required to identify and convincingly demonstrate the signaling function of the transporter.

In higher eukaryotes, evidence for transporters functioning as transceptors has also been obtained, such as the amino acid transporter SNAT2 in mammalian cells [88], *Drosophila*'s high-affinity amino acid sensor PATH [89], the plant nitrate transporter NRT1.1 [22], the plant ammonium transporter AMT1;3 [24], and the mammalian GLUT2 glucose transporter [25]. In all these cases, modification of the transporter caused effects that could not be explained by the transport function (alone), and were therefore interpreted as indicating a signaling capacity. For instance, separate overexpression of the middle intracellular loop of GLUT2 in a wild-type mouse disturbed the control of food intake by the hypothalamus. It was even suggested that the signaling function of GLUT2 may constitute an interesting drug target for treatment of eating disorders and associated metabolic diseases [25]. About half of all currently used drugs act on receptor proteins. As drug targets, they present important advantages compared to intracellular proteins. Transceptors therefore may constitute a new appealing class of drug targets, especially for metabolic diseases. Ligand-induced receptor internalization is a well-known mechanism for preventing overstimulation of receptor-controlled signaling pathways [55]. Hence, it appears likely that mechanisms that prevent overstimulation of the pathway also operate in transceptor-controlled signaling pathways. Similar to the mechanisms discovered for Gap1, they may tightly control the level of the transceptor protein in the plasma membrane and keep part of the transceptor in an intracellular storage system for rapid delivery to the plasma membrane. Hence, the existence of sophisticated controls on the trafficking of a nutrient transporter in higher eukaryotes may serve as a guide for the discovery of nutrient transceptors in these much less genetically tractable organisms.

## Conclusions and perspectives

The discovery of a signaling function in transporters was surprising, but not really unexpected in light of previous suggestions that receptor proteins may be evolutionarily derived from transporters [45]. Given the importance of precise control of signaling pathways and in the first place of the initiator of the signaling, the receptor, it is not surprising that these proteins are subject to sophisticated regulation of their intracellular localization and trafficking. The fact that similarly sophisticated controls have been discovered for the first well-established examples of eukaryotic nutrient transceptors with transport capacity, in particular the Gap1 amino acid transceptor, supports the hypothesis that at least part of this sophisticated regulation has to do with its signaling function as a transceptor. In this paper we have tried to show that certain controls exerted on Gap1 can be interpreted more easily in view of its signaling function rather than its transport function.

It has been difficult to convincingly distinguish the signaling function from the transport function in transceptors. It will be equally difficult to convincingly ascribe the importance of the intracellular trafficking controls on transceptors to either the transport or the signaling function of the protein. What is sure, however, is that all studies on intracellular trafficking of nutrient transporters from now on will have to take into account the possibility that the transporter may not just be a nutrient permease but also a nutrient receptor, and therefore that part of the trafficking controls may have to do with a previously unanticipated signaling function of the transporter.

## Abbreviations

MVB: multivesicular body
PKA: protein kinase A
TGN: trans-Golgi network
